# A good creator should be a wise listener: how user feedback impacts the performance of user-generated videos

**DOI:** 10.3389/fpsyg.2026.1810744

**Published:** 2026-07-20

**Authors:** Chunxue Mu, Chenwei Li, Qianzhou Du, Xinyu Wang, Weiguo Fan

**Affiliations:** 1 Southeast University, Nanjing, China; 2 Department of Intelligent Operations and Marketing, Xi’an Jiaotong-Liverpool University, Suzhou, China; 3 Faculty of Business for Science and Technology, School of Management, University of Science and Technology of China, Hefei, China; 4 School of Management, University of Science and Technology of China, Hefei, China; 5 Department of Business Analytics, The University of Iowa, Iowa City, IA, United States

**Keywords:** componential theory of creativity, information overload theory, performance of user-generated video, text analysis, user feedback

## Abstract

**Introduction:**

User-generated video performance underpins the sustainable development of the user-generated video economy, yet its influencing factors remain underexplored. Drawing on the componential theory of creativity and information overload theory, this study examines how user feedback correlates with video performance and whether creator experience moderates such relationships.

**Methods:**

Empirical analyses were performed based on Bilibili user-generated video data. We classified user feedback by quantity and type and adopted regression models to test the hypothesized moderating effects.

**Results:**

User feedback volume exhibited a significant inverted U-shaped association with video performance. Creator experience positively moderated the relationship’s turning point, elevating the critical feedback volume threshold. Additionally, different feedback types showed heterogeneous correlations with video performance.

**Discussion:**

This study refines the nonlinear mechanism and boundary condition of audience feedback affecting creators’ creative output, enriching relevant theoretical research. It provides differentiated operational and managerial implications for video platforms to optimize content ecology and sustainable development.

## 1 Introduction

With the flourishing development of social media platforms, user-generated videos (UGVs) have gained increasing popularity among consumers. UGVs, referring to videos produced by non-professionals, have become a powerful marketing tool influencing viewers’ decisions (Chang et al., 2019; Liu-Thompkins and Rogerson, 2012). Many video sharing platforms have emerged in recent years and are widely embraced. For example, on YouTube, a large number of video creators consistently upload content on the platform, contributing 3.7 million videos per day^1^. On Bilibili, the number of monthly active users has reached over 348 million by December 2024. According to a recent report, the revenue of global online video platform market is estimated to be valued at $17.2 billion by 2026 and continue to maintain good growth momentum^2^.

Video sharing platforms encourage different types of video creators to output high quality content to achieve high-level user engagement. This success can be attributed to the differentiated competition mode adopted by online platforms, often described as a “flywheel effect,” which is driven by a professional user-generated ecosystem and two-sided network effects (Rong et al., 2019). Diversified creators could create high quality original videos, which may bring in mass viewers, thus attracting more high-level video creators and creating a stable and sustainable environment for content generation (Yang et al., 2024). High-performing creators can attract significant attention, generate substantial business value for sponsoring enterprises (Zhao et al., 2021), and earn valuable incoming traffics and monetary rewards (Dong et al., 2020; Dost et al., 2019; Huang et al., 2019). Therefore, video creators must embrace their viewers for better performance. In such a creative environment, the performance of UGVs becomes the lifeblood of the user-generated video economy, determining not only the growth and competitiveness of video sharing platforms but also the degree of viewers’ engagement and interaction (Goes et al., 2014). Therefore, enhancing the performance of UGVs has become a critical issue to be addressed for sustaining the stable and healthy development of user-generated video economy.

Existing studies on UGV performance have extensively explored various influencing factors and yielded abundant findings. For instance, improvements in the video creators’ social network structures (Fang et al., 2023; Mattke et al., 2020; Susarla et al., 2012), video content (Hautz et al., 2014; Wang et al., 2022), and technical attributes (Hautz et al., 2014) could significantly promote the video performance. Personal characteristics of video creators and their interactions with viewers also correlate with audience’s decision to click a “like” button or follow an account of the video creator (Wang S. A. et al., 2022). Interestingly, performance driven by creativity (Hossain and Islam, 2015; Liu et al., 2022) provides an innovative perspective to improve the current work on the performance of UGVs. However, the role of creativity in correlating with video performance remains underexplored. Considering feedback is a powerful tool to enhance the performance of individual creativity (Mattern et al., 2013), this study investigates how user feedback is associated with UGV performance from the innovative perspective of creativity.

It is necessary to clarify that, in this study, user feedback acts as an external stimulus to adjust creators’ creative thinking and behaviors, and then correlates with the creative output of their new videos. On video-sharing platforms, ordinary audiences cannot directly evaluate the abstract creativity level of works. Instead, they intuitively judge content novelty, practicality and quality, and express their recognition through likes, favorites and coins. Therefore, these interactive indicators are adopted to reflect the market performance of creators’ creative output, rather than measuring creativity itself.

Feedback can increase individuals’ creative output by improving their professional skill and intrinsic motivation (Zhou, 1998). It also assists content understanding, facilitates high-quality content production, and motivates content creators (Grant and Berry, 2018; Jabr et al., 2014; Tang et al., 2021), contributing to the performance of UGVs. However, previous research on feedback is inadequate, lacking a comprehensive and contextualized understanding in the context of user-generated videos. To address this gap, this study introduces two key attributes of user feedback (Liu et al., 2022)—quantity and type—to examine their associations with on UGV performance. Moreover, to capture the heterogeneity of video creators, this study incorporates creators’ experience as a moderator, which may significantly correlate with their creative output. Specifically, this study addresses the following research questions:

*RQ1*: How does the quantity of user feedback relate to the performance of user-generated videos on video sharing platforms?

*RQ2*: How does the type of user feedback relate to the performance of user-generated videos on video sharing platforms?

*RQ3*: How does video creators’ experience moderate the association between user feedback and the performance of user-generated videos?

This study mainly replenishes the existing literature in three aspects. First, it investigates the association of user feedback, a typical external factor of individual creativity, on the performance of UGVs. This work not only extends the componential theory of creativity into the video-sharing economy, but also clarifies the link between audience feedback, creators’ creative output, and observable UGV performance. Second, supplementing the existing literature, this study examines other important attributes of user feedback, such as the quantity of feedback, the type of feedback, and the heterogeneity of feedback recipients, in the context of video sharing platforms to provide a comprehensive contextualized understanding. Third, it empirically analyses the nonlinear association between the amount of user feedback and the performance of UGVs. Prior researchers have over-stressed the benefits of large amount of user feedback. However, information overload is a problem which should not be ignored especially in the era of information explosion. This study supplements the previous literature by taking information overload into consideration and refines the present understanding of the feedback mechanism.

## 2 Literature review

This section reviews two main streams of relevant literature, which are the performance of user-generated videos and user feedback.

### 2.1 Performance of user-generated videos

Researchers have long been interested in the performance of UGVs. Many influencing factors of UGV performance have been well documented. For example, well designed video content could attract viewers’ attention and gain popularity (Hautz et al., 2014; Wang N. et al., 2022; Wang et al., 2020). The technical aspects of a video, such as the resolution, sharpness, brightness and contrast, contribute to its online performance (Hautz et al., 2014). The interactive tactics employed and flow experience provided by the promotion strategy could help attract more viewers. Social capital and social interactions of video creators, such as relationship herd, social network structures, social presence and endorsement, also significantly increase the number of searches and the rate of diffusion for a video, and improve its chance of success (Fang et al., 2023; Hsu, 2020; Mattke et al., 2020; Susarla et al., 2012). Besides, individuals’ motivation to view Danmaku videos (Chen et al., 2017) and creators’ characteristics (Liu-Thompkins and Rogerson, 2012) are important to video performance as well.

Interestingly, some scholars are interested in the performance driven by creativity and have considered performance from the angle of creativity (Hossain and Islam, 2015; Liu et al., 2022). Creativity determines the novelty and quality of video content, and further influences audience responses and UGV performance. Performance differences among UGVs can be regarded as the external reflection of creators’ creative output, which provides an innovative perspective for relevant research. Different views on creativity have been documented. Amabile (1988) believed that creativity is a novel and useful idea produced by a small group of people working together. Sternberg and Lubart (1998) defined creativity as “the ability of an individual to produce novel ideas or products of practical value.” It has also been pointed out that communication and interaction with others would enhance creativity (Ford, 1996). Although these definitions and findings still hold in the age of social media, the internal and external influencers of creativity might have changed. The new characteristics of communications and interactions in the rapid changing social commerce environment should be considered. Therefore, it is of greater importance to understand the performance correlated with creativity in a social environment. It should be noted that creativity is a comprehensive construct that novelty, usefulness, quality are all necessary conditions for a piece of content to be considered creative (Liu et al., 2022). All these conditions may contribute to the performance of user-generated videos; thus, creativity is a core driver of video performance, so applying creativity theories helps explain the mechanism of UGV performance.

### 2.2 User feedback

Feedback is generally defined as the information gap between the actual state and the ideal state (Ramaprasad, 1983). It also serves as “structural or procedural knowledge to help users interpret, reformulate, and reframe the problem related to the idea generation—implementation processes” (Liu et al., 2022), referring to an individual’s creative output comparing with predetermined criteria. Feedback can be used to assist content understanding, facilitate high-quality content production and motivate content creators (Grant and Berry, 2018; Jabr et al., 2014; Tang et al., 2021), and contribute to the quality of user-generated videos. For instance, Davidson and De Stobbeleir (2011) found that employees’ perceptions of the feedback environment may affect their creativity performance. Also, feedback could mediate the relationship between employees’ perceived organizational support or work-related stress and their creativity performance (De Stobbeleir et al., 2011; Hon et al., 2013). Therefore, user feedback plays a vital role in optimizing creators’ creative output, and ultimately correlates positively with the improvement of UGV performance.

Much of the literature has explored the association of user feedback with performance in various contexts, and revealed that user feedback is beneficial to relationship building, consumer decision-making, and the prosperity of user-generated content (Huang et al., 2019; Liu et al., 2022; Wang S. A. et al., 2022; Yan et al., 2023). Specifically, the associations of user feedback with outcomes depend on the valence of feedback (Hossain and List, 2012), message framing of feedback (Huang et al., 2019), and the timing, source, and form of feedback (Yan et al., 2023). However, few scholars have examined other important attributes of user feedback to provide a holistic understanding and extended to the context of video sharing platforms. In this study, we supplement the existing literature by taking the quantity of feedback, the type of feedback, and the heterogeneity of feedback recipients into consideration.

## 3 Theoretical foundation

On video-sharing platforms, creators adjust their content creation strategies according to user feedback, which further correlates with the performance of new videos. Audiences intuitively recognize the performance through likes, favorites and coins. Therefore, these interactive indicators are used as proxy variables to reflect the practical effect of creators’ content output.

### 3.1 Componential theory of creativity

According to the componential theory of creativity, an individual’s creativity is influenced by both internal and external factors (Amabile, 1983). Internal factors include expertise (e.g., factual knowledge and technical skills in the relevant domain), creative thinking skills (e.g., cognitive process that facilitates the formation of new thinking) and intrinsic task motivation (e.g., interest, enjoyment or a sense of personal challenge). External factor refers to the surrounding environment, which acts as a stimulus for creativity. Past research has mostly focused on the impact of the internal factors of creativity and yielded rich results (Amabile et al., 1996). User feedback, as one of the most important factors in the external environment, plays a crucial role in the content generation on social media platforms. However, most previous research on the componential theory of creativity have acknowledged the value of interactions between content creators and viewers (Manca and Ranieri, 2016; Susarla et al., 2012; Yang et al., 2019) and overlooked the importance of user feedback, as a major form of interaction and a representative external factor, for individual creativity, which will be examined in our study.

While the componential theory of creativity has been widely used to explain individual innovative behaviors and organizational innovations (Chae et al., 2023), it has not been used to explain the mechanism of how user feedback correlates with individuals’ creative output. Therefore, this study aims to examine the association of user feedback, as a typical external factor, with the output of creators’ creativity in the context of user-generated video creation. Specifically, we introduced two important attributes of user feedback (Liu et al., 2022), e.g., quantity and type, to examine the association of user feedback with the performance of UGVs. Although feedback facilities idea generation and implementation, video creators may suffer from information overload induced by excess feedback received (Liu et al., 2022), correlating negatively with their creativity performance. Meanwhile, considering the differentiation of creativity performance effected by the type of feedback (Sijbom et al., 2018; Wang et al., 2015), we follow the previous work (Lee et al., 2014), which adopted information-based feedback (e.g., technical or numerical feedback) to assess user-generated content in a relatively rational and objective way and relationship-based feedback (e.g., personal feelings or emotions) to assess content in a more subjective way, and propose a dichotomization of user feedback consisting of content-related feedback and emotion-related feedback (Lee et al., 2014; Liu et al., 2022; Wang et al., 2020).

### 3.2 Information overload theory

Information overload, which is also known as cognitive overload, refers to situations in which the amount of information is too large that people are unable to process it in time and fail to extract and assimilate the ideas conveyed in the information (Robinson and Bawden, 2009). In other words, there is a limit to the amount of information that people can absorb and process at one time. When the limit is exceeded, “overload” occurs and hinders people’s decision-making (Benselin and Ragsdell, 2016). Hargittai et al. (2012) have identified that time sensitivity, decision need, information structure and information quality are all incentives for information overload.

Indeed, an important reason for information overload lies in the individuals’ ability to process information (Pee et al., 2020), which may vary from person to person. If an individual has a high information processing ability, he/she is more likely to solve problems and make decisions timely and is less likely to be affected by information overload. The information processing ability not only depends on a person’s learning capacity but is also influenced by one’s experience in the relevant field. However, few scholars have considered information overload caused by overwhelming feedback as well as the potential impact of individuals’ ability to process information. Therefore, in this study, we introduce “experience” to represent the information processing ability of user feedback and to explain how feedback overload correlates with the performance of UGVs. As one of the important antecedents of individual creativity, it has been documented that video creators’ past experience in video-making may significantly correlate with the individual creativity performance (Guo et al., 2017). Thus, it is appropriate to introduce “experience” as the moderator between use feedback and the performance of UGVs.

In general, the componential theory of creativity sets up the scaffolding for this study to understand the association of user feedback (external influencing factor of creativity) with video performance (performance of creativity) in the context of video sharing platforms with the personal traits of video creators (internal influencing factor of creativity) controlled. Information overload theory introduces and explains the moderating effect of experience (heterogeneity of video creator) on the correlational link between user feedback and video performance. These two theories cooperate with each other to provide a more comprehensive understanding of the performance of UGVs correlated with video creators’ creativity.

## 4 Research framework

### 4.1 Research model

This study investigates the association of user feedback with the performance of UGVs moderated by video creators’ experience. The amount of user feedback and the type of user feedback, including content-related feedback and emotion-related feedback, are examined. It should be mentioned that, as shown in Figure 1, a video creator would receive various feedback after the videos are released, and the feedback may correlate with the performance of upcoming videos generated by the video creator.

**Figure 1:**
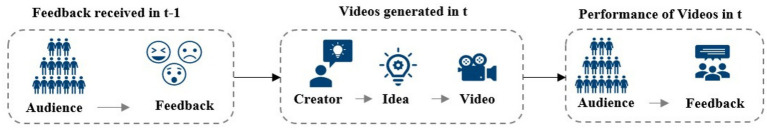
The pattern of feedback-driven video generation.

**Figure 2:**
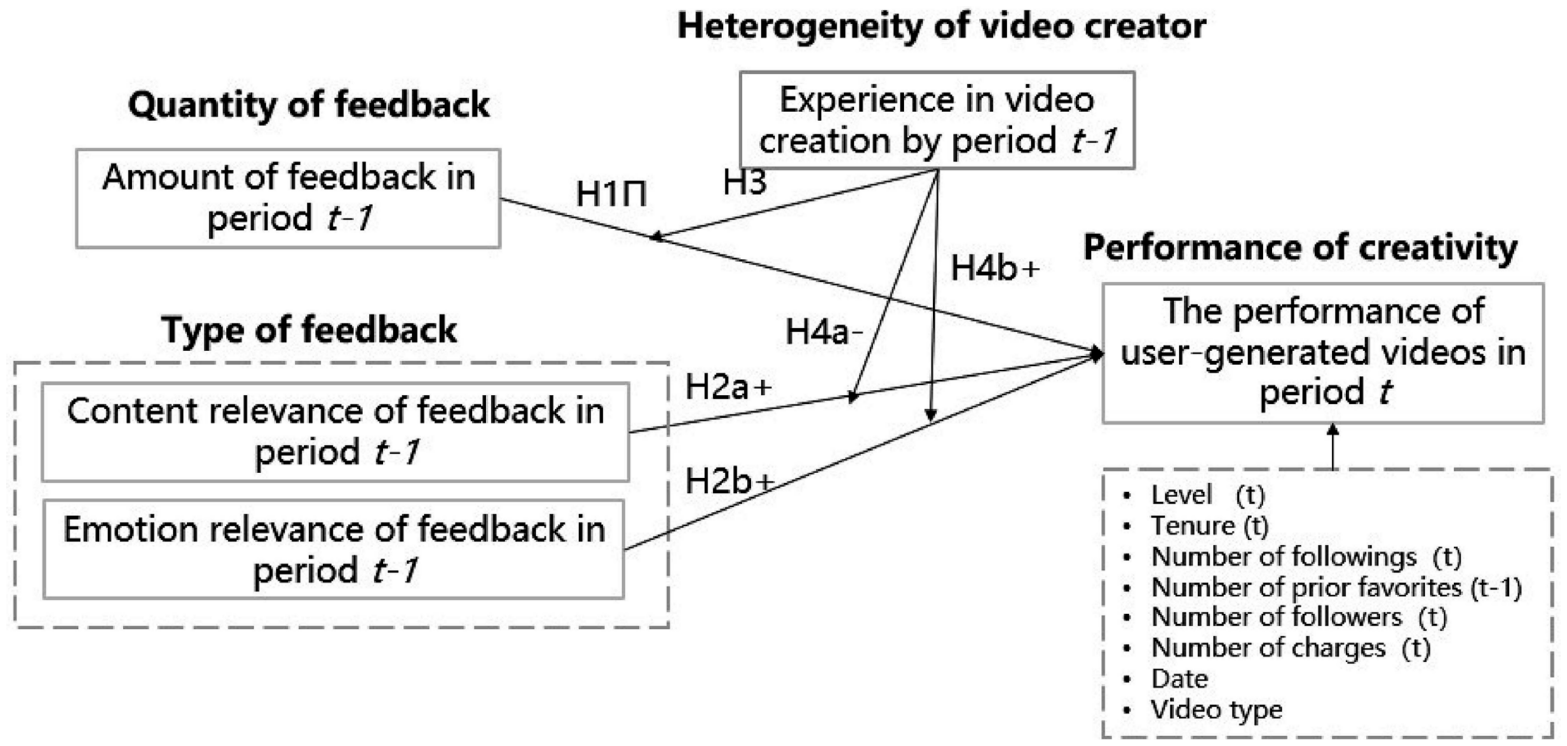
Proposed research model.

Correspondingly, we take this pattern of user feedback obtained from video sharing platforms into consideration and propose the model as shown in Figure 2.

### 4.2 Research hypothesis

#### 4.2.1 The impact of the amount of user feedback

On video sharing platforms, viewers can comment on videos, which allows creators to receive a large amount of user feedback after launching a video. Video creators can process the feedback on the video and gather viewers’ opinions, thus the feedback provides inspiration and materials for the creators to produce better videos (Davidson and De Stobbeleir, 2011). However, if the amount of user feedback exceeds a limit, there is a risk of “information overload” due to the overwhelming amount of feedback received (Case et al., 2005; Robinson and Bawden, 2009). If that is true, video creators are relatively inefficient in extracting the superfluous information which exceeds their processing ability. Thus, they may only make good use of the information before the maximum processing amount to improve the performance of videos in the next period, and fail to get the most effective information for internalization after the maximum processing amount. Therefore, we posit:

*H1*: The amount of user feedback has an inverted U-shaped correlation with the performance of user-generated videos in the next period.

#### 4.2.2 The impact of the type of user feedback

Viewers providing feedback on a specific video implies that viewers have watched the video and taken an interest in the content of the video, thus they would like to express their opinions and interact with the video creator. After receiving the feedback regarding to the content, video creators are usually cognitively stimulated and their degree of contribution to online community may increase (Ogink and Dong, 2019). It has been found that content-related user feedback correlates positively with the likelihood of subsequent ideas being adopted by viewers (Liu et al., 2022). When creators receive more user feedback related to the content, the performance of their subsequent videos will also be increased. Therefore, we posit:

*H2a*: The degree of content relevance in user feedback is positively associated with the performance of user-generated videos in the next period.

User-generated videos can be seen as a special form of communication, where video creators desire to get feedback from others and are eager for a sense of belonging within certain communities (Park and Lee, 2021). For video creators, especially those novices who have little attention from the viewers in the early stages, they desire positive responses from viewers to derive enjoyment from content generation and to be more engaged with their work. If the videos are recognized and praised by most viewers (i.e., responses with positive sentiment take up a higher proportion in the feedback), they will be more motivated to learn the skills related to video generation and improve the performance of videos in the next period as a response, which forms a virtuous circle. Therefore, we posit:

*H2b*: The degree of emotion relevance in user feedback is positively associated with the performance of user-generated videos in the next period.

#### 4.2.3 The impact of experience

So far, we have predicted the direct effects of user feedback volume on creators’ subsequent UGV performance. Prior research indicates that individuals differ substantially in information searching, processing and utilization capabilities, which further shapes their behavioral responses (Mishra et al., 2015). In this study, we utilize the total number of uploaded videos to measure experience, which reflects creators’ long-term production activity on the platform rather than professional competence per se. Continuous content creation over time gradually cultivates creators’ stable habits of screening, interpreting and leveraging user feedback. Creators with higher experience are more adept at extracting useful inspiration from audience comments, so the positive contribution of feedback to creative output becomes more prominent. Besides, creators with rich experience grow familiar with community norms and gradually build stronger resilience against information overload. When facing massive feedback, they can efficiently distinguish valuable opinions from redundant information and mitigate the negative impacts of excessive comments. As a result, the inverted U-shaped relationship between feedback volume and UGV performance will shift its inflection point to the right because the adverse effects of information overload only emerge when feedback reaches a higher volume. Thus, we posit:

*H3*: With an increase of creators’ experience, the turning point of the inverted U-shaped effect of the amount of user feedback on the performance of user-generated videos in the next period shifts to the right.

As creators keep producing content and gain higher experience, they gradually form fixed content positioning and mature creative rhythms (Wang et al., 2020). For these creators, content-related feedback offers limited incremental value for further creative optimization (Ogink and Dong, 2019). Diverse and scattered content suggestions are difficult to integrate into their well-established creation frameworks, which restricts the improvement of content quality and video performance. Meanwhile, long-term high-frequency content production may lead to creative burnout and reduced intrinsic motivation (Dong et al., 2020). In this context, emotion-related feedback such as audience praise and encouragement plays a vital role in lifting creators’ spirits and sustaining their creative enthusiasm. Accordingly, experience weakens the positive effect of content-related feedback while amplifying the beneficial impact of emotion-related feedback on creative output and UGV performance. Thus, we posit:

*H4a*: Creators’ experience weakens the positive association between the degree of content relevance in user feedback and the performance of user-generated videos in the next period.

*H4b*: Creators’ experience strengthens the positive association between the degree of emotion relevance in user feedback and the performance of user-generated videos in the next period.

## 5 Methodology

### 5.1 Data description

Our data was crawled from Bilibili.com using Python crawler completed by Huoshaoyun,^3^ which is a professional data analysis company focusing on text, picture and video sharing platforms. In the first step, all the relevant information of video creators in seven primary categories on Bilibili, including food, domestic creation, games, knowledge, life, fashion, film & TV, was obtained. Then, for each of these categories, we selected the most active 3,000 video creators measured by their number of published videos over the past 3 months before April, 1st 2021. Among them, 1,000 video creators were randomly chosen to build the creator pool. In the third step, all the related information both about these 1,000 video creators and the videos they have created between April 1st, 2021 and June 30th, 2021 were collected. Finally, after removing the creators who were inactive or not original creators (only copying videos from others) in the designated period, we have 571 video creators in total, together with the information about all the 7,637 videos generated by them. Other information, such as the number of daily followers of each creator, the uploading time of each video, the number of likes of each video, the number of coins of each video, the number of favorites of each video, and the viewers’ feedback, etc., were collected as well. During data cleaning, we removed reposted, fully imitative videos and clickbait content relying on sensational effects, to avoid the interference of non-original and provocative content on empirical results.

### 5.2 Variables

#### 5.2.1 Dependent variable

**Figure 3:**
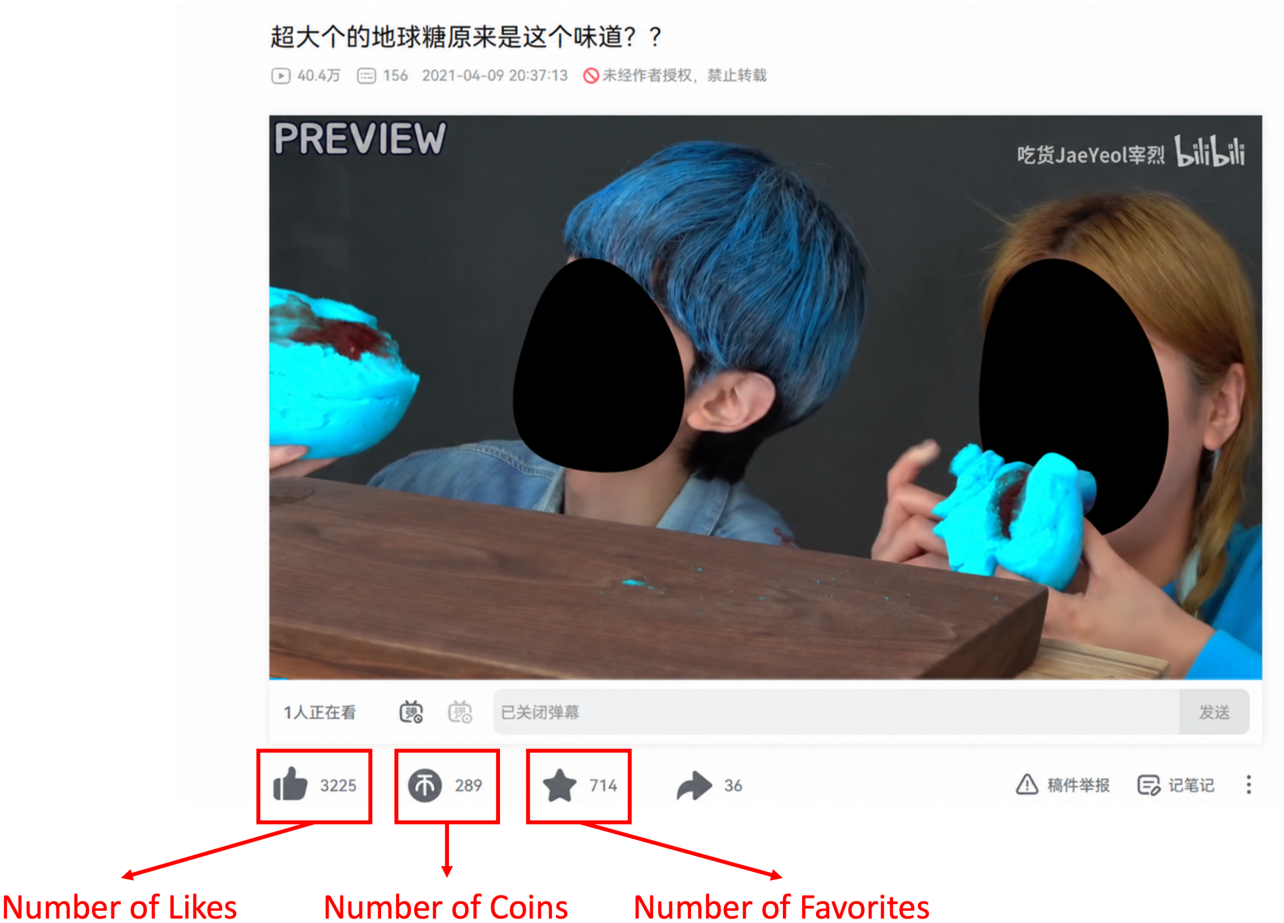
Performance indicators on Bilibili.

The dependent variable in this study is the performance of UGVs. Three indicators are obtained from Bilibili, including the number of favorites, the number of likes, and the number of coins, to reflect the performance of UGVs (see details in the Figure 3). These indicators are appropriate because they mainly reflect viewers’ intuitive feelings toward the video. Referring to existing studies (Burtch et al., 2022; Hautz et al., 2014; Liu et al., 2022; Tang et al., 2021), the number of favorites, likes and coins are adopted to measure video audience interaction performance. These indicators reflect audience recognition of video content and serve as behavioral proxies for content output in the UGV ecosystem.

Among them, the number of favorites is relatively a more suitable indicator to measure the performance of UGVs. First, “favorite” means that the viewers are interested in the content of videos and would like to continue watching or recommend it to their friends or family members in the future (Ma et al., 2019; Susarla et al., 2012). Second, compared to the number of coins which may be contributed by crazy fans, the number of favorites is relatively objective and is not easily manipulated by extrinsic motivation. Third, “favorite” targets at the videos directly and it expresses a recognition of video content rather than other attributes of video creator. Therefore, we use the number of favorites to measure the performance of UGVs. It should be noted that the number of these indicators gradually stabilized down 7 days after the release of a video. Thus, we choose 7 days as the window period in the data analysis.

#### 5.2.2 Independent variables

*Amount of feedback*: Here, we use the number of comments received by each video in period *t* − 1 to represent the amount of user feedback. For example, if one video obtains 50 comments in period *t* − 1, its amount of feedback takes the value of 50.

*Content relevance*: Content relevance refers to the degree to which each viewer comment is associated with the core content of a video. We calculate this indicator by measuring the cosine similarity between individual viewer comments and the full text of the corresponding video. The full text of a video integrates three sources of information: video title, video descriptive text, and audio content converted to text via speech-to-text (STT) technology. We adopted a standardized text preprocessing pipeline before similarity calculation. First, we used the Jieba Chinese segmentation tool to split all video full texts and user comments into separate phrases. Then, we removed punctuation marks, symbols and stop words based on the Harbin Institute of Technology Chinese stop word list, and only retained valid noun phrases. Lastly, we constructed word bag vectors for the processed video text (Vector **A**) and each comment (Vector **B**) respectively. Specifically, this study adopted cosine similarity to quantify content relevance as shown in Equation 1:


$$\text{similarity}=\cos(\theta )=\frac{A*B}{A*B}\frac{\sum_{i=1}^{n}A_{i}*B_{i}}{\sqrt{\sum_{i=1}^{n}{\left(A_{i}\right)}^{2}}*\sqrt{\sum_{i=1}^{n}{\left(B_{i}\right)}^{2}}}$$ (1)

here $\;A_{i}$ and $B_{i}$ denote the individual components of vectors A and B , respectively. Vector **A** = word vector of the integrated video text; Vector B = word vector of a single comment. Where Vector **A** stands for the word vector of the integrated full text of one video, and Vector **B** stands for the word vector of a single viewer comment. The cosine similarity for an individual comment ranges from 0 to 1, where a larger value represents stronger topical matching between the comment and video content. After computing a separate similarity score for every comment posted under the same video, we sum all comment-level similarity scores to generate the aggregate content relevance value for that video. These summed content relevance values are reported in Table 1 and used in the subsequent regression analysis.

**Table 1:** Statistics of variables.

Variable	Observations	Mean	SD	Min	Max
Performance of user-generated video	7,122	807.3	3101.7	0	86,338
Amount of feedback	7,122	925.9	2386.3	1	55,435
Content relevance	7,122	18.7	61.4	0.0	975.2
Emotion relevance	7,122	0.3	0.3	−1.0	1.0
Experience	7,122	78.3	69.3	1	447
Level	7,122	5.7	0.7	2	6
Tenure	7,122	1031.4	631.2	56	4,081
Number of prior favorites	7,122	813.4	3228.6	0	86,338
Number of followings	7,122	79.7	152.9	0	1,910
Number of followers	7,122	451418.3	734205.1	431	7,257,235
Number of charges	7,122	1719.3	5854.4	0	35,111
Number of coins	7,122	1410.6	8605.8	0	469,740
Number of likes	7,122	5676.2	16513.3	0	548,837

It is necessary to clarify that the cosine similarity calculated here only captures topical matching between each comment and the core video content. Even a high summed similarity value cannot be equated to high feedback quality, constructiveness or practical utility, since content relevance merely reflects topic overlap. Dimensions such as rational suggestions, logical reasoning and actionable advice are not measured in this study due to research scope and technical limitations.

*Emotion relevance*: Emotion relevance refers to the net sentiment scores of comments received by a video in period *t* − 1 (Huang et al., 2019). We adopted the Linguistic Inquiry and Word Count (LIWC) text analysis tool (Pennebaker et al., 2015)^4^ and its Chinese dictionary^5^ to extract sentiment information from user-generated content. Because LIWC relies on dictionary-based word matching, we first used the Jieba Chinese word segmentation tool to segment the Chinese comments into separate words and phrases before applying the Chinese LIWC dictionary. The segmented comments were then processed by LIWC to identify words belonging to positive and negative emotion categories, based on which we calculated the net sentiment score for each video. Consistent with established LIWC measurement norms in relevant literature (Huang et al., 2019; Zhou et al., 2018), we calculated net sentiment based on LIWC positive and negative scores and constructed a normalized index to operationalize emotion relevance, as shown in Equation 2:


$$\begin{array}{l} \text{Emotion relevance}\\ =\frac{\text{LIWC postive sentiment}-\text{LIWC negative sentiment}}{\text{LIWC positive sentiment}+\text{LIWC negative sentiment}} \end{array}$$ (2)

The value ranges from −1 to 1; a higher value represents stronger positive emotion in user feedback.

#### 5.2.3 Moderating variable

*Experience*: Experience is measured as the total number of original videos a creator has uploaded by period *t* − 1 since registering on Bilibili. This indicator mainly reflects creators’ long-term activity level and practical production accumulation on the platform, rather than directly representing their professional capabilities. Continuous content creation enables creators to gradually form stable habits of screening, analyzing and utilizing user feedback. Compared with novice creators, creators with higher accumulated production experience are more likely to develop routines for screening, interpreting, and using user feedback, which may help them deal with large volumes of comments and reduce the potential negative influence of information overload. For instance, a creator with 20 published videos has richer practical experience in content operation than a novice with only a few works.

#### 5.2.4 Control variables

Considering the correlation of social attributes with the video performance (Fu et al., 2018; Roy et al., 2013), several control variables are included: (a) level of a video creator, indicating the rank shown on Bilibili; (b) tenure of a video creator, indicating how long the video creator has joined Bilibili; (c) number of prior favorites received by a video creator, representing viewers’ recognition for all the videos generated by a video creator by period *t* − 1; (d) number of followings a video creator has, representing a video creator’s intention to actively learn from others; (e) number of followers a video creator has, representing the popularity of a video creator; (f) number of charges received by a video creator, representing the cash reward a video creator has received; (g) uploading date of a video. In this study, if a video was released on April 1, the value of the date will be 1, and so on; (h) video type, which falls into seven primary categories of videos on Bilibili.com including food, domestic creation, games, knowledge, life, fashion, film & TV. Different types of videos are different in terms of their length, pattern, etc. All the variables are summarized in Table 2.

**Table 2:** Summary of variables.

Variable	Definition
Dependent variables	
Performance of user-generated video	Number of favorites a video has obtained in period *t*
Independent variables	
Amount of feedback	Number of comments a video has received in period *t* − 1
Content relevance	The summation of cosine similarity scores of all comments under a video in period *t* − 1, where each score measures the topical matching between a single comment and the video’s integrated full text (title, description, STT audio)
Emotion relevance	The sentiment of comments received by a video in period *t* – 1
Experience	Total number of original videos uploaded by a creator by period *t* − 1; reflects creators’ platform activity level and long-term content production accumulation
Control variables	
Level	The rank of a video creator on Bilibili by period *t*
Tenure	The number of days a video creator has joined Bilibili by period *t*
Number of prior favorites	The number of favorites a video creator has obtained by period *t* − 1
Number of followings	The number of followings a video creator has by period *t*
Number of followers	The number of followers a video creator has by period *t*
Number of charges	The number of charges a video creator has received by period *t*
Date	Release date index ranging from 1 to 91
Video type	One of the seven primary categories a video belongs to

### 5.3 Regression model

Based on the above variables, the research model is presented as the equation below. Here, i represents each video and t represents each period. $\beta_{i}$ is the coefficient of independent variables, $\delta_{j}$ is the coefficient of date dummy variables, $\gamma_{n}$ is the coefficient of video type dummy variables, and ε is the residual error. STATA 15.1 package was used for data analysis. The results are listed and discussed in Section 6.


$$\begin{array}{l} \text{Video Performanc}e_{i,t}|X_{i,t-1}=\beta_{0}+\beta_{1}*\text{Amount of feedbac}k_{i,t-1}\\ +\beta_{2}*\text{Content relevanc}e_{i,t-1}+\beta_{3}*\text{Emotion relevanc}e_{i,t-1}\\ +\beta_{4}*\text{Experienc}e_{i,t-1}+\beta_{5}*\text{Amount of feedbac}k_{i,t-1}*\text{Experienc}e_{i,t}\\ +\beta_{6}*\text{Content relevanc}e_{i,t-1}*\text{Experienc}e_{i,t-1}+\beta_{7}*\text{Emotion relevanc}e_{i,t-1}\\ {}*\text{Experienc}e_{i,t-1}+\beta_{8}*\text{leve}l_{i,t}+\beta_{9}*\text{Number of prior favorite}s_{i,t-1}\\ +\beta_{10}*\text{Number of followin}g_{i,t}+\beta_{11}*\text{Number of follower}s_{i,t}\\ +\beta_{12}*\text{Number of charge}s_{i,t}+\sum_{j=1}^{37}\delta_{j}*(\text{Date}\_\text{dummie}s_{j})\\ +\sum_{n=1}^{7}\gamma_{n}*(\text{video}\_\text{type}\_\text{dummie}s_{n})+\varepsilon \end{array}$$

## 6 Results analysis

Due to the wide range of data variables, a natural logarithmic operation was performed to make the results of regression analysis smoother. Taking the natural logarithm did not change the nature of the data but could help reduce the covariance and heteroskedasticity of the model to some extent (Wooldridge, 2003). The final data set is summarized in Table 1.

### 6.1 Regression results

Table 3 lists the main results of negative binomial regression. Model 1 depicts the nonlinear relationship between the amount of feedback and the number favorites received by a video in period t. Model 2 adds the content relevance and emotion relevance into Model 1. Then, Model 3 adds the interaction terms between content relevance, emotion relevance, and experience, respectively. Model 4 further adds the interaction term between both the linear and squared term of the amount of user feedback and experience.

**Table 3:** Main results of negative binomial regression.

Variable	Model 1	Model 2	Model 3	Model 4	Model 5
Amount of feedback	0.1280^***^	0.0582^***^	0.2009^***^	0.1951^***^	0.1941^***^
	(0.0130)	(0.0182)	(0.0184)	(0.0182)	(0.0186)
Amount of feedback^2^	−0.0762^***^	−0.0855^***^	−0.0418^***^	−0.0384^***^	−0.0444^***^
	(0.0042)	(0.0046)	(0.0051)	(0.0048)	(0.0051)
Content relevance		0.1296^***^	0.1036^***^	0.1219^***^	0.1101^***^
		(0.0196)	(0.0188)	(0.0191)	(0.0194)
Emotion relevance		0.4549^***^	0.3601^***^	0.4252^***^	0.3975^***^
		(0.0528)	(0.0507)	(0.0516)	(0.0515)
Experience			−0.8072^***^	−0.6759^***^	−0.8228^***^
			(0.0267)	(0.0218)	(0.0273)
Amount of feedback * experience			−0.1203^***^		−0.0886^***^
			(0.0104)		(0.0177)
Amount of feedback^2^ * experience			0.0301^***^		0.0383^***^
			(0.0039)		(0.0043)
Content relevance * experience				−0.0784^***^	−0.0377^*^
				(0.0130)	(0.0228)
Emotion relevance * experience				0.1694^***^	0.2552^***^
				(0.0565)	(0.0578)
Constant	−4.3287^***^	−4.1723^***^	−2.7471^***^	−2.8303^***^	−2.7637^***^
	(0.4424)	(0.4397)	(0.4223)	(0.4239)	(0.4219)
Control variables (level, followers, etc.)	Control	Control	Control	Control	Control
Date effect	Control	Control	Control	Control	Control
Video type	Control	Control	Control	Control	Control
Observations	7,122	7,122	7,122	7,122	7,122
Log-likelihood	−47107.4	−47054.4	−46644.5	−46691.1	−46635.8
Pseudo *R*-squared (McFadden)	0.1272	0.1282	0.1358	0.1349	0.136

*p < 0.1; **p < 0.05; ***p < 0.01. DV, the number of *favorites* received by a video in period *t*.

**Figure 4:**
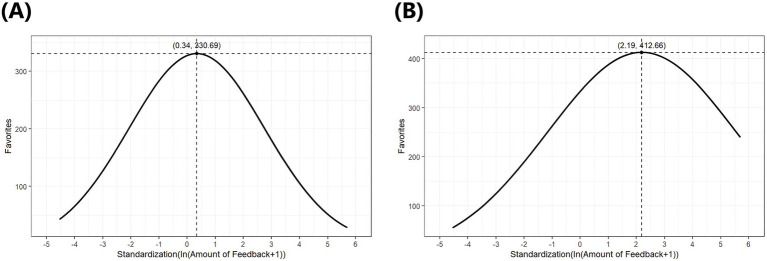
The inverted U-shaped curve between the amount of user feedback and the performance of user-generated video in period *t*. **(A)** The inverted U-shaped curve between the amount of user feedback and the performance of user-generated video in period *t* based on the results of Model 2. **(B)** The inverted U-shaped curve between the amount of user feedback and the performance of user-generated video in period *t* under the moderation of experience based on the results of Model 5.

In Model 2, the coefficient of the squared term of the amount of user feedback is significantly negative (*β* = −0.0855, *p* < 0.01) and the coefficient of the linear term of the amount of user feedback is significantly positive (*β* = 0.0582, *p* < 0.01). This trend remains stable in all the other four models. Combining both the second-order and first-order coefficients of the amount of user feedback, it is easy for us to depict a U-shaped relationship between the amount of user feedback and the performance of UGV in the next period. Within the data range, an extreme point is identified, suggesting a turning point exists in the relationship. The estimated abscissa value of this extreme point is 0.34. It indicates that when the natural logarithmic transformed value of the amount of user feedback is 0.34, the regression curve attains its maximum value. Thus, H1 is supported. The nonlinear relationship between the amount of user feedback and the performance of UGV is displayed in Figure 4A.

Model 2 also provides baseline evidence for Hypotheses 2a and 2b. The coefficient of content relevance is significantly positive (*β* = 0.1296, *p* < 0.01), which reveals that higher topical matching between audience comments and video core content corresponds to better subsequent UGV performance. This result holds consistently across Model 3, Model 4 and Model 5, so H2a is fully supported. Meanwhile, the coefficient of emotion relevance is significantly positive (*β* = 0.4549, *p* < 0.01) in Model 2, demonstrating that more positive net sentiment embedded in user feedback corresponds to favorable values of creators’ future video performance. This significant positive association remains robust in all subsequent full models, which confirms H2b.

The interaction effect between the amount of user feedback and experience are presented in Model 5. Under the moderating impact of experience, it can be observed from Model 5 that, the value of extreme point is 2.19. It indicates that an increase in the experience leads to a rightward shift in the extreme point (from 0.34 to 2.19). Notably, the value of 0 is not encompassed within this confidence interval, indicating a significant effect of experience on the movement of the extreme point. Thus, H3 is supported. The nonlinear relationship between the amount of user feedback and the performance of UGV under the moderation of experience is displayed in Figure 4B.

Based on Model 2 and Model 5, the coefficient of the interaction term between degree of content relevance and experience is significantly decreased (*β*_content relevance * experience_ = −0.0377, *p* < 0.1), supporting H4a. That is, as the experience of video creators increases, the positive relationship between the degree of content relevance in user feedback and the performance of UGVs is weaken (*β* = 0.1101–0.0377 = 0.0724 < 0.1036, *p* < 0.01). The coefficient of the interaction term between the degree of emotion relevance and experience is significantly increased (*β*_emotion relevance_ * _experience_ = 0.2552, *p* < 0.1), supporting H4b. It indicates that experience strengthens the impact of the degree of emotion relevance on the performance of UGVs in the next period (*β* = 0.3975 + 0.2552 = 0.6527 > 0.4252, *p* < 0.01).

Besides, the regression results show that experience has a significant negative main effect on subsequent UGV performance. Intuitively, creators with more published works are expected to produce better-performing videos, so this outcome is somewhat unexpected. We propose that creative fixation is a potential cause (Chrysikou and Weisberg, 2005). Creators with high experience tend to repeatedly adopt mature content frameworks and creative routines formed in previous works, which reduces the novelty of new content. An alternative explanation is that senior creators usually own a large fan base, and new works face higher audience expectations, leading to a gradual drop in the marginal interaction volume of single videos. It is also possible that long-term continuous content production may trigger creative burnout, which reduces creators’ enthusiasm and content quality. It should be noted that these explanations are speculative inferences based on existing theories and industry observations. Our current dataset only consists of behavioral indicators and we cannot empirically verify which mechanism dominates this negative effect.

Lastly, an interesting finding about control variables is that the number of charges was found to be significantly positively correlated with the performance of videos across all the four models. It indicates that monetary incentive is an effective stimulus correlated with improved performance of videos.

### 6.2 Supplementary analysis

To test the possible multicollinearity between variables, we conducted a VIF analysis on all the regressors and the results are listed in Table 4. All the values are less than 5 with the maximum of 3.89. Thus, it can be concluded that the multicollinearity is not of major concern in the model.

**Table 4:** VIF analysis.

Variable	GVIF	D*f*	GVIF^[1/(2*D*f*)]^
Amount of feedback	4.351	1	2.086
Content relevance	3.019	1	1.738
Emotion relevance	1.151	1	1.073
Experience	1.410	1	1.187
Level	1.805	1	1.344
Tenure	1.603	1	1.266
Number of followings	1.315	1	1.147
Number of followers	2.369	1	1.539
Number of charges	1.733	1	1.316
Date index	1.532	93	1.002
Video type	2.118	7	1.055

**Figure 5:**
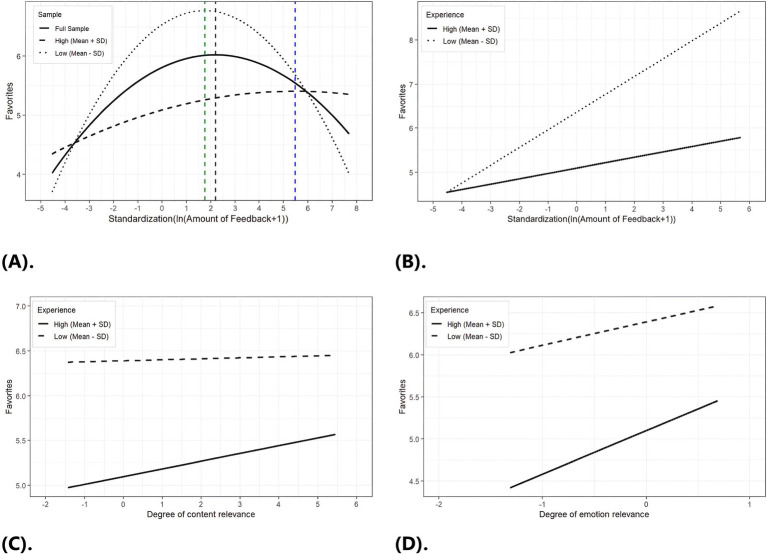
The marginal plots of the moderation effect of experience. **(A)** The moderation effect of experience on the inverted U-shaped relationship between the amount of user feedback and the performance of UGVs. **(B)** The moderation effect of experience on the linear relationship between the amount of user feedback and the performance of UGVs. **(C)** The moderation effect of experience on the relationship between content relevance in user feedback and the performance of UGVs. **(D)** The moderation effect of experience on the relationship between emotion relevance in user feedback and the performance of UGVs.

Besides, to better demonstrate the moderation effects of experience, the marginal plots are provided in Figure 5. The moderation effects of experience (high experience vs. low experience) are significant across all the relationships, and the related moderating hypotheses (H3, H4a, H4b) are all supported.

### 6.3 Robustness check

To test the reliability of the main results, we conducted robustness check using the number of coins and the number of likes, respectively, as measures for the performance of UGVs, and re-ran the data analysis using negative binomial regression. Besides, we also tested the main results with OLS regression. Table 5 lists the negative binomial regression results of the number of likes, Table 6 lists the negative binomial regression results of the number of coins, and Table 7 list the OLS regression results of the number of favorites.

**Table 5:** Robustness check (1) using negative binomial regression.

Variable	Model 1	Model 2	Model 3	Model 4	Model 5
Amount of feedback	0.3447^***^	0.2672^***^	0.3865^***^	0.3848^***^	0.3833^***^
	(0.0106)	(0.0148)	(0.0153)	(0.0149)	(0.0154)
Amount of feedback^2^	−0.0761^***^	−0.0858^***^	−0.0540^***^	−0.0546^***^	−0.0549^***^
	(0.0034)	(0.0037)	(0.0041)	(0.0039)	(0.0042)
Content relevance		0.1214^***^	0.1021^***^	0.1114^***^	0.1055^***^
		(0.0160)	(0.0154)	(0.0156)	(0.0160)
Emotion relevance		0.0829^*^	0.0323	0.0629	0.0521
		(0.0426)	(0.0414)	(0.0422)	(0.0423)
Experience			−0.6042^***^	−0.5567^***^	−0.6086^***^
			(0.0211)	(0.0176)	(0.0216)
Amount of feedback * experience			−0.0452^***^		−0.0306^**^
			(0.0085)		(0.0145)
Amount of feedback^2^ * experience			0.0110^***^		0.0142^***^
			(0.0032)		(0.0036)
Content relevance * experience				−0.0366^***^	−0.0194
				(0.0106)	(0.0184)
Emotion relevance * experience				0.0908^**^	0.1115^***^
				(0.0414)	(0.0424)
Constant	−0.3933	−0.3983	0.6612^*^	0.5473	0.6507^*^
	(0.3570)	(0.3559)	(0.3440)	(0.3444)	(0.3441)
Control variables (level, followers, etc.)	Control	Control	Control	Control	Control
Date effect	Control	Control	Control	Control	Control
Video type	Control	Control	Control	Control	Control
Observations	7,122	7,122	7,122	7,122	7,122
Log-likelihood	−61191.0041	−61161.3147	−60818.7094	−60827.4444	−60815.9454
Pseudo *R*-squared (McFadden)	0.1301	0.1305	0.1354	0.1352	0.1354

*p < 0.1; **p < 0.05; ***p < 0.01; DV, the number of *likes* received by a video in period *t*.

**Table 6:** Robustness check (2) using negative binomial regression.

Variable	Model 1	Model 2	Model 3	Model 4	Model 5
Amount of feedback	0.4390^***^	0.2713^***^	0.5281^***^	0.5114^***^	0.5180^***^
	(0.0141)	(0.0196)	(0.0190)	(0.0187)	(0.0191)
Amount of feedback^2^	−0.0956^***^	−0.1142^***^	−0.0475^***^	−0.0526^***^	−0.0496^***^
	(0.0047)	(0.0050)	(0.0053)	(0.0051)	(0.0054)
Content relevance		0.2684^***^	0.2552^***^	0.2789^***^	0.2681^***^
		(0.0210)	(0.0194)	(0.0196)	(0.0200)
Emotion relevance		0.5286^***^	0.4556^***^	0.5121^***^	0.4936^***^
		(0.0592)	(0.0541)	(0.0554)	(0.0553)
Experience			−1.1330^***^	−1.1090^***^	−1.1437^***^
			(0.0275)	(0.0225)	(0.0281)
Amount of feedback * experience			−0.0904^***^		−0.0567^***^
			(0.0111)		(0.0185)
Amount of feedback^2^ * experience			0.0029		0.0095^**^
			(0.0041)		(0.0046)
Content relevance * experience				−0.0868^***^	−0.0482^**^
				(0.0135)	(0.0235)
Emotion relevance * experience				0.1742^***^	0.1917^***^
				(0.0612)	(0.0626)
Constant	−5.6420^***^	−5.6126^***^	−2.8673^***^	−2.9711^***^	−2.8839^***^
	(0.4863)	(0.4800)	(0.4424)	(0.4420)	(0.4417)
Control variables (level, followers, etc.)	Control	Control	Control	Control	Control
Date effect	Control	Control	Control	Control	Control
Video type	Control	Control	Control	Control	Control
Observations	7,122	7,122	7,122	7,122	7,122
Log-likelihood	−46573.0901	−46467.6264	−45700.2454	−45701.8073	−45695.0163
Pseudo *R*-squared (McFadden)	0.1432	0.1451	0.1593	0.1592	0.1593

*p < 0.1; **p < 0.05; ***p < 0.01; DV, The number of *coins* received by a video in period *t*.

**Table 7:** Robustness check (3) using OLS regression.

Variable	Model 1	Model 2	Model 3	Model 4	Model 5
Amount of feedback	0.1876^***^	0.0972^***^	0.2584^***^	0.2501^***^	0.2466^***^
	(0.0138)	(0.0192)	(0.0190)	(0.0187)	(0.0191)
Amount of feedback^2^	−0.0519^***^	−0.0637^***^	−0.0094^*^	−0.0133^***^	−0.0124^**^
	(0.0044)	(0.0048)	(0.0052)	(0.0050)	(0.0052)
Content relevance		0.1513^***^	0.1209^***^	0.1562^***^	0.1366^***^
		(0.0207)	(0.0194)	(0.0197)	(0.0200)
Emotion relevance		0.5439^***^	0.4026^***^	0.4917^***^	0.4546^***^
		(0.0550)	(0.0517)	(0.0527)	(0.0524)
Experience			−0.7083^***^	−0.5993^***^	−0.7327^***^
			(0.0274)	(0.0224)	(0.0280)
Amount of feedback * experience			−0.1757^***^		−0.1145^***^
			(0.0106)		(0.0181)
Amount of feedback^2^ * experience			0.0180^***^		0.0298^***^
			(0.0040)		(0.0044)
Content relevance * experience				−0.1723^***^	−0.0861^***^
				(0.0134)	(0.0235)
Emotion relevance * experience				0.2220^***^	0.3111^***^
				(0.0577)	(0.0584)
Constant	−3.8669^***^	−3.7509^***^	−3.0518^***^	−3.1390^***^	−3.0590^***^
	(0.4653)	(0.4606)	(0.4320)	(0.4340)	(0.4309)
Control variables (level, followers, etc.)	Control	Control	Control	Control	Control
Date effect	Control	Control	Control	Control	Control
Video type	Control	Control	Control	Control	Control
Observations	7,122	7,122	7,122	7,122	7,122
*R* ^2^	0.5144	0.5244	0.5851	0.5805	0.5876
Adjusted *R*^2^	0.5069	0.5169	0.5784	0.5737	0.5809

*p < 0.1; **p < 0.05; ***p < 0.01; DV, the number of *favorites* received by a video in period *t*.

## 7 Discussions and contributions

### 7.1 Discussions

This study has obtained some interesting correlational findings. First, the empirical results confirm that the volume of user feedback has a significant inverted U-shaped correlation with UGV performance. Creators are likely to experience information overload when confronted with excessive comments, which correlates negatively with creative output after a threshold. Second, both content-related and emotion-related feedback correlate positively with subsequent video performance, indicating that different types of audience responses bear distinct correlational links with UGC outcomes. Third, creators with richer experience have stronger capabilities to process user feedback and are less vulnerable to information overload. Meanwhile, as experience grows, content-related feedback gradually loses its incremental value, while emotion-related feedback plays a more prominent motivational role.

From a psychological perspective, these statistical patterns can be further explained by creators’ cognition, emotion and motivational changes. Moderate user feedback satisfies creators’ need for social recognition and boosts creative motivation, whereas excessive comments trigger cognitive overload and drain cognitive resources, forming an inverted U-shaped relationship with UGV performance. Creators with greater experience possess more mature cognitive processing abilities, so the inflection point of this curve shifts rightward. In addition, content-related feedback primarily influences creators’ cognition and works better for novice creators; for experienced creators with fixed creative frameworks, such guidance becomes less effective. By contrast, emotion-related feedback alleviates creative burnout and enhances intrinsic motivation, exerting stronger positive effects on long-term creators. Furthermore, prolonged content production tends to cause creative fixation and psychological burnout, which explains the negative main effect of experience on video performance.

### 7.2 Theoretical contributions

The study is expected to contribute to the existing literature in several aspects. First, this work enriches the current research about the performance of UGVs. Under the guidance of the componential theory of creativity, we investigate the association of user feedback, a typical external factor of individual creativity, with the performance of UGVs. It not only extends the componential theory of creativity into the video sharing economy, but also deepens the understanding of how audience feedback correlates with creators’ creative output.

Second, this study supplements the existing literature by taking the quantity of feedback, the type of feedback, and the heterogeneity of feedback recipients into consideration. Much of the literature has explored the beneficial impacts of user feedback on performance in various contexts (Huang et al., 2019; Liu et al., 2022; Wang S. A. et al., 2022; Yan et al., 2023). However, the findings are limited and inadequate, which cannot provide a comprehensive contextualized understanding of user feedback. We have examined other important attributes of user feedback to provide a holistic understanding and extended to the context of video sharing platforms.

Third, this study supplements the previous literature by taking information overload into consideration and empirically analyzing the nonlinear impact of the amount of user feedback on the performance of UGVs. Prior researchers have given more emphases on the benefits of large amount of information. Our work reveals that there is an invert U-shaped relationship between the amount of user feedback and the performance of UGVs. When the amount of user feedback exceeds the optimal point, it may become a burden for video creators, thus should be given special attention.

Besides, existing studies about user feedback have overwhelmingly employed survey methodology and relied heavily on self-reported data and few of them explored user feedback in the field of video sharing platforms (Chiu et al., 2006; Park and Lee, 2021). This work collects secondary data from Bilibili to examine the impact of user feedback on the performance of UGVs. Meanwhile, the moderation effect of video creators’ experience on the relationships between the various attributes of user feedback and the performance of UGVs is also tested in this work, perfecting the present understanding of the feedback mechanism.

### 7.3 Practical implications

This study is of value to video sharing platforms practitioners with insights. To begin with, the understanding of user feedback in this study can be applied to other platforms to improve the utilization of user feedback. For example, the viewers can be suggested to post comments in a more specific way, e.g., to offer advice on the video content or to express their feelings and emotions. This act not only allows feedback from viewers to be presented in a more organized way, but also reduces the burden for the platform and the video creators to process the feedback.

Next, information overload should not be ignored. To address this problem, video sharing platforms can adopt strategic information processing, e.g., machine learning and text mining, to assist video creators in filtering out irrelevant or worthless feedback, and aggregate similar feedback to reduce the stress and anxiety of processing excessive information for video creators. It also contributes to the sustainable development of video sharing platforms and assist video creators in enhancing their content-generation capabilities.

Then, it is found that the experience of video creator plays an important moderating role between the quantity and type of feedback and the performance of videos, thus a differentiated management strategy for video creators with different experience should be executed. For experienced uploaders, they need emotional incentives, such as encouragement and acknowledgement, to motivate them to devote into further innovative creation. While for novices, substantive feedback, especially suggestions and comments related to content, could help produce high quality videos and earn good performance. Therefore, some training courses to help video creators become familiar with the platform and tips for video creation would be useful.

Last but most important, the results about user feedback offer video creators a relatively objective way to understand their strengths and find their deficiencies. In the past, when video creators were caught in a vortex of information overload, they cannot identify the value and begin to doubt their abilities. This study reminds the video creators to be with a critical attitude toward information (Kwon et al., 2007) which is to have a good understanding of the information world and to treat information critically. When facing with large amount of user feedback, video creators should develop critical thinking and take advice wisely.

### 7.4 Limitations and future research

This study leverages an empirical research approach to explore the impact of user feedback on the performance of UGVs and the moderating impact of experience. However, some aspects still need to be improved. The current data set is obtained from Bilibili, with relatively longer videos than other video sharing platforms, such as Tiktok (Jiang et al., 2024). Future study could be conducted on short video platforms and test whether video length would affect the results.

Meanwhile, other possible factors, such as the graphic clarity, the sound effect of videos, the payment information to boost the video views, the advertising strategy for video promotion, and the system recommendation algorithm adopted by the platform, are not consider in this study due to the technical constraints or data inaccessibility. Future research could include more factors into consideration and obtain richer results.

Besides, the current data set only contains videos released in 3 months. It would be better if larger and longer data set could be obtained and analyzed to increase the generalizability. The comparison of performance in different periods based on larger and longer data set could also bring more insights to understand the evolution process of video performance.

In terms of variable measurement, we adopt the total number of uploaded videos to measure creators’ experience. This single indicator mainly reflects platform activity level and production frequency, and cannot fully capture multi-dimensional connotations of experience, such as professional video-making skills, information processing literacy and content quality. Meanwhile, we use cosine similarity to measure content relevance of user feedback, which cannot comprehensively evaluate the quality, constructiveness and practical value of feedback. Future research can construct a multi-dimensional experience index by combining multiple indicators, including account tenure, historical content performance, platform certification and vertical domain familiarity, to achieve more accurate measurement, and combine manual content coding, sentiment analysis and fine-grained text mining to evaluate multi-dimensional attributes of feedback.

Moreover, for text sentiment analysis, we used the Chinese dictionary of LIWC for measurement. Although we supplemented Bilibili-specific internet vocabulary into the Chinese LIWC dictionary, dictionary-based sentiment measurement still has inherent limitations when processing rapidly updated internet slang, homophones and implicit rhetorical expressions unique to the platform. New internet memes emerge rapidly, which may cause minor recognition errors. Future research can adopt large-scale pre-trained Chinese language models (e.g., BERT, RoBERTa) trained on social media corpus to achieve more accurate fine-grained sentiment analysis.

Lastly, it is also one of our limitations that the current study focuses more on the analysis of correlations rather than causality. The study cannot conclusively demonstrate that feedback itself causes improvements in future video performance. Besides, we observe a negative main effect of experience on UGV performance. Although we put forward several potential explanations including creative fixation and creative burnout, these conjectures cannot be verified by the current data. Future research could try to reveal the causality between user feedback and the performance of UGVs and to uncover the deeper mechanisms underlying the observed patterns.

## Data Availability

The raw analytical dataset supporting the conclusions of this article will be made available by the authors upon reasonable reader request, without undue reservation. The original raw video data sourced from Bilibili cannot be shared due to platform data access restrictions and relevant user privacy constraints.

## Footnotes

1
https://techjury.net/video/how-many-videos-are-uploaded-to-youtube-a-day/

2
https://scoop.market.us/online-video-platform-statistics/

3
https://www.hsydata.com

4 LIWC2015 Operation Manual: https://liwc.app/static/documents/LIWC2015%20Manual%20-%20Operation.pdf

5 LIWC Dictionary Repository: https://www.liwc.app/dictionaries
